# Understanding clients, providers and the institutional dimensions of irrigation services in developing countries: A study of water markets in Bangladesh

**DOI:** 10.1016/j.agwat.2019.05.038

**Published:** 2019-08-01

**Authors:** Khondoker A. Mottaleb, Timothy J. Krupnik, Alwin Keil, Olaf Erenstein

**Affiliations:** a Socioeconomics Program (SEP), International Maize and Wheat Improvement Center (CIMMYT), Carretera Mex-Veracruz, Km. 45, El Batan, Texcoco, CP 56237, Mexico; b Sustainable Intensification Program, CIMMYT, Dhaka, Bangladesh; c SEP, CIMMYT, New Delhi, India; d SEP, CIMMYT, Mexico

**Keywords:** *Boro* rice, Client farmer, Groundwater, Human and social capital, Irrigation service provision, Payment method, Risk, Surface water, Sustainability

## Abstract

Bangladesh is one of the most densely populated nations that nonetheless has largely achieved staple self-sufficiency. This development has been enabled in part by the rapid proliferation of small-scale irrigation pumps that enabled double rice cropping, as well as by a competitive market system in which farmers purchase water at affordable fee-for-service prices from private irrigation pump owners. Excess groundwater abstraction in areas of high shallow tube-well density and increased fuel costs for pumping have however called into question the sustainability of Bangladesh’s groundwater irrigation economy. Cost-saving agronomic methods are called for, alongside aligned policies, markets, and farmers’ incentives. The study assesses different institutions and water-pricing methods for irrigation services that have emerged in Bangladesh, each of which varies in their incentive structure for water conservation, and the level of economic risk involved for farmers and service providers. Using primary data collected from 139 irrigation service providers and 556 client-farmers, we empirically examine the structure of irrigation service types and associated market and institutional dimensions. Our findings demonstrate that competition among pump owners, social capital and personal relationships, and economic and agronomic risk perceptions of both pump owners and farmers significantly influence the structure of irrigation services and water pricing methods. Greater competition among pump owners increases the likelihood of pay-per-hour services and reduces the likelihood of crop harvest sharing arrangements. Based on these findings, we explore policy implications for enhancing irrigation services and irrigation sustainability in Bangladesh.

## Highlights

• Private led irrigation expansion contributed to achieve Bangladesh’s food security.
• Different irrigation payment methods have emerged with expansion of irrigation.
• Social capital, personal relationships and risks shape irrigation payment method.
• Competition among pump owners ensures socially desirable payment method.
• Economic and agronomic risks however influence crop sharing payment method.

## 1 Introduction

Bangladesh faced considerable food shortage challenges in the early 1970s, its post-independence era with a population of less than 70 million (Dorosh, 2000; Hossain, 2009). Bangladesh’s population has more than doubled since (164.7 million in 2017), (GoB, 2018) making it the 8^th^ largest populace on a relatively modest land mass (93^rd^ among 215 countries in terms geographic size), and hence ranks among the top five most densely populated countries (World Bank, 2019a). Yet, the country is now almost self-sufficient in staple food production (Ahmed et al., 2000; Dorosh, 2000; Hossain, 2009; Hossain et al., 2007, 1994; Mottaleb et al., 2015). This achievement comes despite low and declining per capita arable land due to population pressure, and competition for other land uses (United Nations, 2017). Domestic rice and wheat production have more than doubled from an yearly average of 14.1 million metric tons (MMT) during 1971–1990 to 37.4 MMT in 2017–18 (BBS, 2018a, 2017; BRRI, 2019a).

The rapid adoption of modern high-yielding varieties (HYV) along with the proliferation of irrigation, has underpinned this success. According to BRRI (2019a), in 1971–72, with a national average yield 1.28 ton/ha, total paddy rice produced in Bangladesh was 11.1 MMT from 9.3 million ha of cultivated land, in which the contribution of high-yielding rice varieties was 16.1% (1.8 MMT) (BRRI, 2019b). In contrast, in 2017-18, with a national average yield 3.0 ton/ha, total paddy production in Bangladesh was 36.3 MMT from 11.6 million ha of land. This is 226% more than productivity levels observed in 1971-72, with the contribution of high-yielding and stress tolerant cultivars increasing to 94.3% (34.2 MMT) (BRRI, 2019b). During 1971–2018, the total rice area of Bangladesh increased by 25% from 9.3 million ha in 1971–72 to 11.6 million ha in 2017–18 (BRRI, 2019b). The implications of these changes on food self-sufficiency are widely documented (Bera and Kelley, 2002; Dorosh, 2000; Hossain, 1988; Hossain et al., 2012, 2007, 2006, 2003; Mottaleb et al., 2015). In contrast, the contribution of small-scale irrigation systems in Bangladesh has received relatively less attention, although it has been perhaps as equally important as the introduction of new varieties in the enhancement of cereal productivity in Bangladesh, as elaborated below.

After independence in 1971, the Bangladesh government started investing heavily in irrigation, and until 1979, ground and surface water pumping was largely government managed. Original emphasis was on pumping surface and ground water, with the Bangladesh Agricultural Development Corporation (BADC) supplying surface water irrigation pumps, establishing deep tube wells (DTWs) and subsidized fuel to farmer groups and individuals (Mottaleb et al., 2017). Small-scale pump irrigation only rapidly expanded when the government privatized the irrigation facilities and liberalized agricultural machinery imports (Hossain, 2009; Mottaleb et al., 2017). Entrepreneurial farmers acquired irrigation pumps for their own land, and then provided irrigation services to the neighboring farmers on a service-for-fee basis.

Irrigation services and pricing can vary substantially across and within locales (Aggarwal, 2007; Dinar and Subramanian, 1997; Garrido and Calatrava, 2010; Johansson et al., 2002; Rhodes and Sampath, 1988; Sampath, 1992; Wichelns, 2010). Bangladesh is no exception, and over time, different irrigation services, payments methods, and institutions have developed in rural economies (Chowdhury, 2012; Rahman et al., 2015). Payments for irrigation services are now primarily monetized, but in-kind payments through crop sharing are still common in parts of the country. Such service provision arrangements reduce monetary outlays for farmers and provide risk sharing mechanisms (Kajisa and Sakurai, 2005). Monetized payments include fixed and variable rates that may or may not include fuel cost sharing by farmers. Irrigation services that imply real marginal costs (e.g., volumetric irrigation water pricing and/or extra fuel costs by farmers) likely incentivize farmers to use water more efficiently and on a need-based basis (Dinar and Subramanian, 1997; Easter and Liu, 2005). These, therefore, appear to be more environmentally and socially desirable methods. Irrigation services and efficiency become particularly important against the backdrop of declining per capita availability of renewable internal freshwater resources throughout the developing world (World Bank, 2019a).

Bangladesh is primarily a downstream delta – located in the eastern lower Indo-Gangetic Plains (IGP), a relatively fertile plain area that is intensively cultivated. Declining groundwater tables are particularly prominent in the drier northwestern IGP (Erenstein and Thorpe, 2011), and are only occasionally reported in specific locations of the more humid and flood prone eastern IGP in Bangladesh (Qureshi et al., 2015). The total freshwater withdrawal in Bangladesh in 2008 was 35.9 billion m³, of which 88% was used for crop irrigation (World Bank, 2019b). Out of Bangladesh’s 8 million hectares of cropland, 67% (5.37 M ha) is under irrigation, of which 77% is derived from groundwater abstraction (BADC, 2015). Irrigation is particularly prominent for winter season ‘*boro*’ rice, with farmers applying 500–1000 l of water per kg of grain produced (Bouman, 2009). Despite being primarily a river delta with recurrent flooding, the massive extraction of groundwater has resulted in gradually declining water tables in some locales, particularly in Rajshahi Division and to some extent in Khulna Division by between 0.01–0.05 m yearly (Dey et al., 2017; Shamsudduha et al., 2009). Given the high population density and intensively double-cropped rice systems, these indications of declining groundwater tables are an increasing concern in parts of the country. This calls for water-saving agronomic methods, alongside aligned policies, markets, and farmers’ incentives. The study thereby assesses different institutions and water-pricing methods for irrigation services that have emerged in Bangladesh. We examine the factors that affect varying irrigation payment methods in Bangladesh in order to better inform incentives and policies to efficiently use irrigation water. To our knowledge, this is the first empirical attempt to examine the factors that influence types of irrigation contract choice between irrigation service providers and client-farmers in Bangladesh.

## 2 Evolution and expansion of mechanized irrigation in Bangladesh

Aspiring to achieve food self-sufficiency, the government of Bangladesh initially heavily invested in agricultural mechanization (Hossain, 2009; Justice and Biggs, 2013; Mottaleb et al., 2017). During the early ‘green revolution’ of the 1960s, farmers were encouraged to cultivate dwarf rice varieties, apply fertilizer, and irrigation. To expand the irrigated area, the government first established centralized irrigation systems, from which the ground-water based deep tube wells (DTWs) and surface-water based low-lift pumps (LLPs) were supplied to farmers’ groups and cooperatives on rental basis. Until 1978, under the control of the BADC, the government also supplied fuel for pumping at a 75% subsidized rate (Hossain, 2009). By 1978, a total of 9000 DTWs and 35,000 LLPs were reportedly managed by BADC (iDE, 2012).

**Fig. 1:**
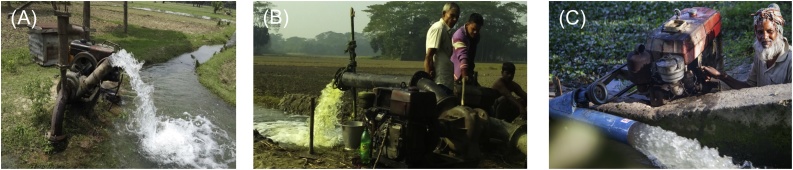
Some irrigation modalities in Bangladesh, including (a) diesel-driven shallow groundwater tube well (groundwater based, Faridpur district, Dhaka division); (b) low-lift surface water pump (Barishal district, Barishal division), (c) an axial-flow surface water pump (Bhola district, Barishal division).

Sources: Photos a and b are by the authors. Photo c is credited to R. Martin.

In the early 1980s, Bangladesh undertook market liberalization policies (Gisselquist et al., 2002; Justice and Biggs, 2013; Mottaleb et al., 2017). Earlier in 1970, in order to reduce the economic and operative burdens, in BADC began selling DTWs and LLPs to farmers’ cooperatives and individual farmers. Over time, the latter group became some of the original pump service providers (Hossain, 2009). The proliferation of pump sets used for irrigation in Bangladesh, however, accelerated in 1989, after the removal of several tariff and non-tariff barriers on the imports of agricultural machinery, including diesel engines (Justice and Biggs, 2013; Mottaleb et al., 2017). During that time, the ban on the imports of small horsepower engines and other agricultural machinery by the private sector, particularly from China, was removed (Justice and Biggs, 2013; Mottaleb et al., 2017). Restrictions of minimum distances between shallow tubewells (STWs) to limit over-abstraction were also abolished (Hossain, 2009). These actions accelerated the proliferation of the private-led small-scale irrigation system in Bangladesh (Fig. 1). The shift to service provision for irrigation was also observed in other South Asian countries – particularly India – and more recently in parts Sub-Saharan Africa where groundwater irrigation is feasible (Diao et al., 2017; Takeshima et al., 2013).

In 1982-83, Bangladesh relied on a mix of STWs, LLPs and DTWs to irrigate 1.52 million hectares of land (Table 1). The total rice and wheat productions were 13.63 and 1.10 MMT, respectively. In contrast, in 2015–16, the irrigated area had increased nearly 5-fold to 5+ million hectares of land – with a more than 10-fold increase in irrigation pumps, particularly STW’s (Table 1). Average cropping intensity (the number of crops harvested from the same land per year) reached 194% in 2017–18 (BBS, 2018b), with total rice, maize and wheat production in 2015–16 registered at 36.3, 3.02 and 1.09 MMT, respectively (BBS, 2019, 2018b; BRRI, 2019a). Bangladesh is now self-sufficient in rice production. Combined with increased economic growth, this has resulted in a decline in extreme poverty (Hossain, 2009).

**Table 1:** Selected irrigation, cropping intensity, and cereal production indicators in Bangladesh since 1982.

Year	No. of irrigation pumps (‘000 units)^1^			Irrigated area (10^6^ ha)^1^	Cropping intensity (%)^3^	Production major cereals (10^6^ MT)		
	DTW	STW	LLP			Paddy rice^5^	Maize^6^	Wheat^7^
1982-83	13.8	93.1	35.5	1.52	150	14.1	<0.01	1.07
1984-85	16.9	147.0	37.0	1.77	152	14.6	<0.01	1.46
1989-90	22.6	260.0	51.0	2.58	168	17.7	<0.01	0.89
1994-95	26.7	488.9	57.1	3.11	175	16.8	<0.01	1.24
1999-00	23.5	707.6	58.1	3.56	176	23.1	0.01	1.84
2000-01	23.2	865.2	71.3	3.77	177	25.1	0.06	1.68
2004-05	27.2	1,129.0	99.3	4.79	177	25.2	0.35	0.98
2009-10	32.9	1,425.1	150.6	5.22	181	32.0	0.89	0.98
2012-13	35.3	1,523.6	170.6	5.37	190^4^	33.8	1.55	1.25
2015-16	36.7	1,517.2	162.4	5.49^2^	194^4^	35.1	2.44	1.35^2^
2017-18	na	na	na	5.59^2^	na	36.3	3.02	1.15^2^

Compiled from: (BADC, 2015, 2013)^1^; (GoB, 2018)^2^; (BRRI, 2019c)^3^; (BBS, 2018b)^4^; (BRRI, 2019a)^5^; (FAO, 2019)^6^, (BBS, 2018a)^7^. Default is column-wise source, unless otherwise indicated. Note: DTW = Deep tube well; STW = Shallow tube well; LLP = Low lift pump.

^na^ indicates data not available.

With the expansion of groundwater abstraction, different forms of pump ownership and management have emerged. Chowdhury (2012) described five types of irrigation systems in Bangladesh, including (1) traditional methods (e.g., swing basket), (2) government managed and centralized canal irrigation projects, and decentralized (3) LLPs, (4) STWs, and (5) DTWs. Initially, the government provided considerable subsidies to establish DTWs. At present, the establishment of DTWs is primarily private, with individuals and/or groups of farmers or cooperatives owning and managing DTWs command areas. In contrast, as a STW establishment is relatively less costly, most STWs are owned by individuals, relatives, and /or friends. Owners of STW pumps usually enter into informal contractual agreements with client-farmers for irrigation services, with the size of the command area determined by the engine capacity, as well as mutual agreements between client-farmers and service providers.

Different forms of irrigation water pricing and payment methods have subsequently emerged. Following the decentralization of BADC’s control over tube wells and LLPs, the primary payment method for irrigation water that emerged was the sharing one-fourth of the crop harvested by farmers with pump owners (Chowdhury, 2012; Rahman et al., 2015). Payment methods have evolved over time towards different forms of monetary systems. These include cash payment per hour of pumping or a seasonal flat-rate basis depending on crop type. Payment methods and the amount of payment per hectare still vary greatly even within small geographic areas (Rahman et al., 2015), although volumetric pricing is rare (Krupnik et al., 2015).

## 3 Conceptual framework and model specification

### 3.1 Conceptual framework

We hypothesize that irrigation water markets consisting of a pump owner and farmer-clients settle on an irrigation water payment method that tends to maximize the profit of the pump owner, while also minimizing farmers’ costs for purchasing irrigation services. These actors’ ability to achieve such a mutually agreeable equilibrium payment method is likely to be associated with the negotiation power of the pump owner and client-farmers, in addition to other environmental, market and exogenous factors. In a functional form, a mutually agreeable irrigation water payment method ${PM}_{i}^{*}$ between pump owner (*p*) and client-farmers (*f*) can be represented as:

(1) $$PM_{i}^{*}=\zeta {PM}_{pf}^{e}\left({NP}_{f}^{*},\;{NP}_{p}^{*},\;\gamma_{j},\;I_{v},\;C_{r}\right){+ϛ}_{i}$$

Where, ${PM}_{i}^{*}$ is the method of irrigation service payment that was chosen in the current season, determined based on the interaction of the negotiation power of the pump owner ${NP}_{p}$ and the negotiation power of the client-farmer $\;{NP}_{f}$, that maximizes pump owner profits (${ᴨ}_{p}^{*})$, and minimizes farmers’ irrigation costs ($C_{f}^{*})$ within an irrigation command area. $PM_{pf}^{e}$ is conversely the payment method practiced in the previous year for the same crop. Note that, because $PM_{pf}^{e}$ is the payment method for irrigation decided in the previous year, a new payment method, ${PM}_{is}^{*},$ is likely to be decided only through the interaction of both the pump owner and client-farmers if the preexisting payment method $,\;PM_{pf}^{e}$, cannot ensure profit maximization and cost minimization for pump owners and farmers, respectively. The factor $\gamma_{j}$ includes environmental and domain variables, such as the competition among pump owners, which can be captured by the density of irrigation service providers in a village, and water availability in the cropping season, while $I_{v}$ includes irrigation-scheme specific characteristics such as the quality of field drainage. Arrow (1968), and Otsuka and Hayami (1988) assert that the presence of a strong patron-client type community relationship can mitigate market failure problems and ensure Pareto optimality. In Eq. (1), the variable $C_{r}$ is the indicator of the community relationship, between the pump owner and the client-farmers. Lastly, ${ϛ}_{i}$ is the random error term.

Several factors can influence the negotiation power of pump owners ${(NP}_{p}^{*})$ and the client famers $\left({NP}_{f}^{*}\right).$ For example, the perceptions of drought or market risk, and willingness to act (irrigate) under different levels of risk, social networks, human and social capital, and physical assets such as landholdings of the pump owner and the client-farmer could influence price negotiation. These factors can influence the negotiation power of the pump owner and the client-farmer, and therefore the irrigation water pricing method. For example, in an ideal environment, a risk-taking farmer may prefer to pay cash to the pump owner on fixed cost-per-hour basis or cash-per-season per unit of land basis, because by doing so, he or she can be the net residual claimer at the end of the rice production season.

In contrast, a risk-taking pump owner might prefer to choose crop-sharing as his or her preferred method, from which they can more reliably claim a portion of grain produced at the end of the season. The degree of human and social capital, and specifically the relationship between pump owners and client-farmers, can also influence negotiation power, and as such the choice of the irrigation payment system. In functional form, this relationship can be described for farmers in Eq. (2):

(2) $${NP}_{f}^{*}=\zeta F_{f}\;\left({HC}_{f},\;R_{f},\;{{SC}_{f},\;SR}_{fp}\right)+{ᵟ}_{i}$$

and for pump owners in Eq. (3):

(3) $${NP}_{p}^{*}=\zeta F_{p}\;\left({HC}_{p},\;R_{p},\;{SC}_{p},\;{SR}_{pf}\right){+\varphi }_{i}$$

In these equations, ${NP}_{p}^{*}$ and ${NP}_{f}^{*}$ are the negotiation power of the pump owner and client-farmer respectively, ${HC}_{p},$ ${HC}_{f},$ are the level of human capital measured by years of formal schooling, and $\;\;R_{p}$ and $\;\;R_{f}$ are the self-assessed risk scores provided by pump owners and farmers, respectively (see Section 3.2). ${SC}_{p}$ and ${SC}_{f}$ are the social capital and physical asset scores of the pump owner and client-farmer; ${SR}_{pf}$ and ${SR}_{fp}$ are an indicator of the social relationship between pump owner and client-farmer and ${ᵟ}_{i}$, and $\varphi_{i}$ are error terms (also described in Section 3.2).

We further hypothesize that the strength of the social relationship between pump owner and client-farmer can vary and is likely to have a considerable degree of influence on the payment method. For example, both pump owner and the client-farmer may be familial relatives, and they could, for example, can reside in the same village, shop in the same markets, and may pray in the mosque. These social interactions and their resulting relationships, therefore, can also influence irrigation water pricing payment methods and fees. As it is infeasible to estimate all three Eqs. (1), (2), (3) separately to identify the factors influencing the method of irrigation payment in a particular location, we developed reduced form functions collapsing Eqs. (1), (2), (3) separately separately for farmer-clients (Eq. (4)) and pump owners (Eq. (5)) as follows:

For client-farmers:

(4) $${PM}_{s}^{*}=\zeta {PM}_{f}^{e}\left({HC}_{f},\;R_{f},\;{{SC}_{f},\;SR}_{fp},\;\gamma_{j},\;I_{v},\;C_{r}\right)+{ϛ}_{i}$$

For pump owners:

(5) $${PM}_{s}^{*}=\zeta {PM}_{p}^{e}\left({HC}_{p},\;R_{p},\;{SC}_{p},\;{SR}_{pf},\;\gamma_{j},\;I_{v},\;C_{r}\right)+\varrho_{i}$$

### 3.2 Empirical model specification and estimation strategy

We operationalize the conceptual framework to identify the personal and social relation factors as well as human capital endowment and environment domain that can affect the mode of irrigation water payment method. The resulting empirical model is specified as follows:

(6) $${PM}_{s}=\beta_{0}+{\left(EV\right)}_{s}\beta_{s}+{\left(HC\right)}_{i}\theta_{i}+{ϓ}_{i}\;{\left(Social\;relation\;index\right)}_{i}+\Lambda_{s}\;({Northern\;region\;dummy)}_{s}+\varepsilon_{i}$$

Where ${PM}_{s}\;$ is the dependent variable that assumes a value of zero if the payment method in an irrigation scheme requiring cash payment seasonally per unit of land by the client-farmer, in addition to the supply of fuel from the farmer. We assume a value one if the payment method is on a cash-per-hour irrigation service basis, or a value of two where farmers pay cash-per-season per land unit, but farmers are not required to supply their own fuel. The model assumes a value of three if farmers share a portion of their harvested crops with the pump owner for irrigation. The default payment method (${PM}_{s}$ = 0) is cash per season with client-farmer supplying fuel.

Explanatory variables include a vector of irrigation scheme specific variables (*EV_i_*) that include:

(1) the number of irrigation service providers (pump owners) operating in a sampled village;
(2) a dummy variable that assumes a value of 1 if the community voluntarily participated in irrigation system maintenance, including within-field canal and/or field drainage operations (zero otherwise);
(3) a dummy variable (value of one) if there was no reported shortage of water in the pump command area at the peak of the *boro* rice season (zero otherwise), and;
(4) a dummy if the command area has a poor water drainage system (value of one) if excessive water accumulates within the irrigation scheme that causes stagnant waterlogging and that can potentially reduce crop productivity (0 otherwise).

The vector of variables *HC_i_* is comprised of pump owner and client-farmer specific variables, including:

(5) familial relative dummies that assume a value of one if the pump owner and client-farmer have at least one blood relative employed in a government sector or who works as or around local politicians (zero otherwise);
(6) At the time of data collection, we asked the sampled respondents on how much risk they usually take in their daily economic activities and asked them to score it in between zero to ten, in which with zero indicating a completely risk-averse attitude of the sampled pump owners and farmer-clients, and 10 indicating a strong preference for risk-taking. The self-assessed general risk scores provided by pump owners and farmer-clients are included in *HC_i_*.

The vector *HC_i_* includes some additional variables for the pump owner and client-farmer:

(7) number of years of schooling;
(8) the household size measured by the number of immediate family members;
(9) area total land cultivated land (ha) during the *boro* season; and
(10) dummy variables that assume a value of one if the major occupation of the pump owner and farmer-client involves off-farm employment (zero otherwise).

In Eq. (6), the independent variable *social relation index_i_* includes the social interaction indicators between a pump owner and client-farmer. This index is constructed by applying the Principal Component Analysis (PCA) to dummy variables for pump owner and client-farmer familiar relations, residence the same village, and for owners and clients who pray in the same mosque or temple. These variables are intended to capture the influence of the social and personal relationships on irrigation water payment method. Details of the process of generating the relation index are included in Appendix A. The *Northern region dummy* is a dummy variable that assumes a value of one (zero otherwise) if the surveyed village was located outside greater Barishal region that is characterized by a higher potential for and use of surface water irrigation (Krupnik et al., 2017) and that tends to be poorer with limited physical infrastructure than other study areas (Mottaleb et al., 2016). $\beta_{0}$ is a scalar parameter and $\beta_{s}$, $\theta_{i},{ϓ}_{i}$, and $\Lambda_{i}$ are the parameters to be estimated; $\varepsilon_{i}$ is the error term. In solving Eq. (6), we applied the multinomial logit estimation, as the dependent variable (payment method type) is categorical and independent. Eq. (6) is estimate separately for pump owner and client-farmers.

## 4 Data sources

### 4.1 Study area and sampling

This study is based on primary data collected from April 22 to June 8 in 2015 from 139 pump owners who provided irrigation services, and from 556 farmer-clients who purchased their irrigation services. In Bangladesh, dry season *boro* rice is the major irrigated crop, although some supplementary irrigation may also provided to the summer/wet season *aus* and *aman* rice and other winter crops, such as wheat or maize (Krupnik et al., 2017; Qureshi et al., 2015). As the primary focus of this study is to examine the factors that affect the payment methods and amount of payment for irrigation services, we focus on the dry season *boro* rice farmers and the irrigation service providers (pump owners) in the completed 2013–14 season.

**Fig. 2:**
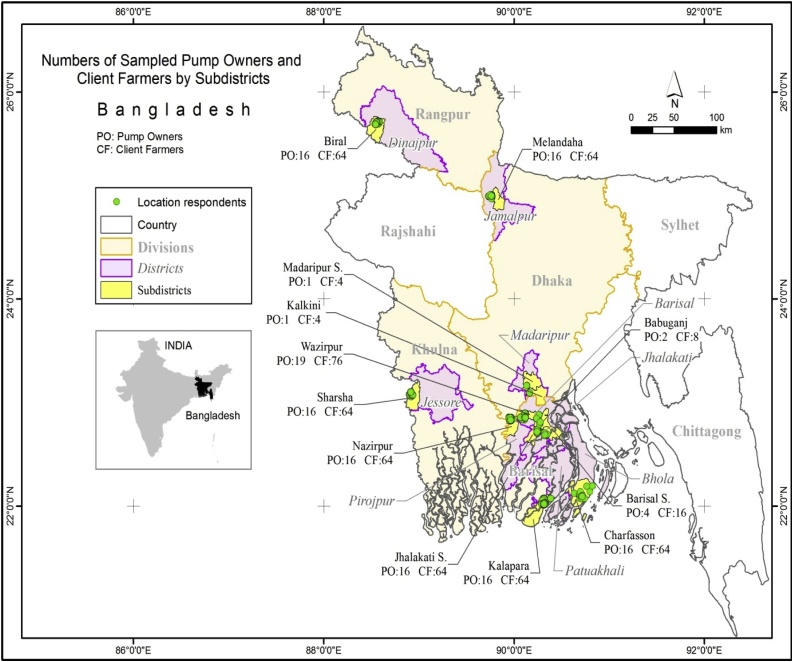
Survey locations and the numbers of sampled pump owners and client-farmers by sampled sub-districts, Bangladesh.

Source: Authors.

We focused on two prevailing smallholder irrigation systems: surface water irrigation using LLPs that are common in Barishal division, and ground-water extraction using STWs, which are more common in north and west (including Dhaka, Rangpur and Khulna divisions, Fig. 2). Pump owners were randomly selected based lists of irrigation service providers supplied by the Department of Agricultural Extension. After selecting the pump owner, we requested them to supply the names of four client-farmers who purchased irrigation services in the 2013–14 winter season. Of 89 pump owners and their 356 clients, farmers were sampled from Barishal division, and a further 50 pump owners and 200 client-farmers were sampled from Dhaka, Khulna, and Rangpur divisions (Table 2). The sample thereby covers four divisions, nine districts, 12 sub-districts, 15 unions, and 43 villages (Fig. 2). According to BADC (2015), out of 1.63 million DWs, STWs and LLPs, 19.3% were electrically powered, with the remainder mainly reliant on diesel. In our sample, out of 139 irrigation schemes, only six used electricity, with the remainder using diesel.

**Table 2:** Boro rice area, pumps, sample distribution, and reported irrigation services by sampled sub-districts, Bangladesh.

Division	District	Subdistrict	Suitable land (‘000 ha) for boro rice^1^	Total nos. of pumps^1^	Sampled respondents (*n*)		% Sampled farmers by irrigation service			
					Pump owners	Farmers	Hourly	Seasonal with client fuel	Seasonal without client fuel	Crop share
Barishal	Barishal	Babuganj	10.80	137	2	8	0	0	0	100
		Barishal sadar	19.2	380	4	16	0	0	0	100
		Wazirpur	19.5	1,089	19	76	36.8	0	47.4	15.8
	Bhola	Char Fasson	29.5	1,120	16	64	0	0	100	0
	Jhalokati	Jhalokati sadar	15.7	856	16	64	0	0	100	0
	Patuakhali	Kalapara	15.3	487	16	64	6.25	87.5	6.25	0
	Pirojpur	Nazirpur	15.4	1,830	16	64	6.25	62.5	31.25	0
Dhaka	Jamalpur	Melandaha	20.2	11,830	16	64	0	0	100	0
	Madaripur	Madaripur sadar	22.6	2,132	1	4	0	0	0	100
		Kalkini	22.4	1,501	1	4	0	0	0	100
Khulna	Jashore	Sharsha	27.5	12,844	16	64	18.75	34.38	46.88	0.
Rangpur	Dinajpur	Birol	13.0	15,067	16	64	6.25	93.75	0	0
Total or Average	9	12	231.2	49,273	139	556	9.35	32.01	50.72	7.91

Sources in addition to the survey: BARC (2019)⁠^1^ and BADC (2013).

### 4.2 Descriptive findings

**Fig. 3:**
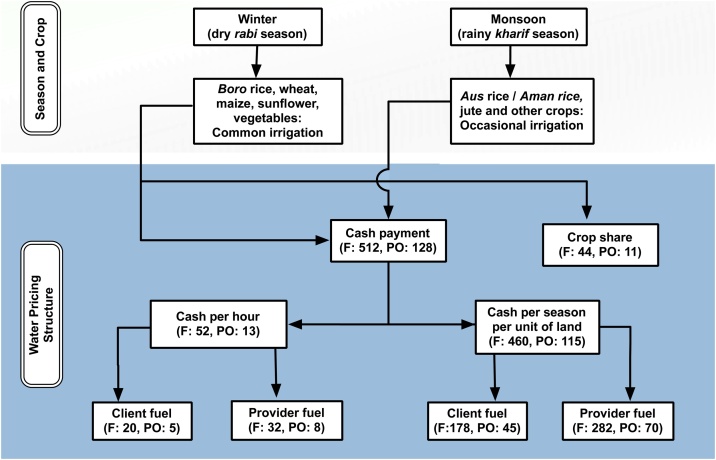
Different types of irrigation service institutions in Bangladesh (numbers indicate the number of observations in each group). F: Client-farmer, PO: Pump owner.

We identified four major irrigation payment methods (Fig. 3) including:

(1) *Hourly payment*, in which farmer-clients pay cash per hour to pump owners (includes both with and without client fuel given our limited sample size),
(2) *Seasonal with client fuel,* in which client-farmers pay a fixed cash rate per season per unit of land, and in addition, supply fuel or provide fuel costs for the full season,
(3) *Seasonal without client fuel*, where farmer-clients pay a flat cash rate per season per unit of land without additional fuel charges, and
(4) *Crop sharing,* also known as share cropping, in which farmer-clients trade an agreed share of harvested grain and/or straw (typically 10–20%) in lieu of cash for irrigation.

All of the sampled irrigation service providers were using the centrifugal pumps and practiced flood irrigation. No solar irrigation pumps were identified, and none of the sampled farmer-clients or irrigation service providers used alternate wetting and drying (AWD) or System of Rice Intensification (SRI) techniques, which was widely promoted but also critiqued in Bangladesh and South Asia, to address the increasing water scarcity in rice-based cropping systems (Bouman and Tuong, 2001; Lampayan et al., 2015). Overall, out of 556 sampled farmers (and 139 service providers), 9% practice the hourly system, 32% used the seasonal system with client-farmer fuel supply, 51% used the seasonal without fuel and 8% crop sharing systems, respectively A total of 512 client-farmers (and 128 service providers) thus followed cash-based payment services–with only 44 sampled farmer-clients (and 11 service providers) using sharecropping (primarily in selected locales, Table 2). A total of 460 farmer-clients (and 115 service providers) followed seasonal payment, of which 282 farmers (and 70 service providers) paid a seasonal flat rate, and the other 178 farmer-clients (and 45 service providers) provided fuel or paid fuel costs. The observed mix payment categories (a base rate plus farmers’ supply or payment per unit of fuel used for irrigation services) can be described as a two-part tariff system (Dinar and Subramanian, 1997; Easter and Liu, 2005), and to some extent incentivize need-based irrigation as farmers can save on fuel costs. The hourly payment service was split into two relatively small subsets, 20 client-farmers who also supplied fuel and another 32 only paying the hourly rate. For simplicity, we clubbed these groups together during analyses.

On average, a client-farmer allocated 0.22 ha land for *boro* rice in 2013–14. Each sampled command areas had < 19 client-farmers with a mean command area of 5.74 ha (Table 3). On average, more than 40% of the sampled client-farmers reported that they participate in preparing within-field canals and drainage systems on an annual voluntary basis. Nearly 77% reported irrigation water was sufficiently available in the 2013–14 *boro* season (Table 3). More than 95% of the sampled pump owners and client-farmers live in the same village, and nearly 74% pray in the same mosque or temple. On average, nearly 35% of the sampled client-farmer and pump owners are relatives (Table 3). On average, 43 pump owners operated in each sample village.

**Table 3:** Descriptive statistics for sampled pump owners and client-farmers by irrigation service, Bangladesh.

	All	Irrigation service				Kruskal-Wallis rank test Chi^2^ (overall differences)
		Hourly	Seasonal		Crop share	
			With client fuel	Without client fuel		
		a	b	c	d	(a ≠ b≠c ≠ d)
Pump owners (*n*)	139	13	44	71	11	
Client-farmers (*n*)	556	52	178	282	44	
*Boro* rice cultivated by farmers (ha)	0.22	0.20^x^	0.22^xy^	0.22^x^	0.15^y^	537.14* (0.10)
Client-farmers per command area (*n*)	18.4	13.5^x^	7.6^y^	16.9^x^	77.7^z^	140.83***(0.00)
Command area (ha)	5.74	5.22 ^xz^	3.80 ^x^	5.36 ^z^	16.68 ^y^	58.89***(0.00)
Farmer participation in-field canal and drainage maintenance (%)	40.3	53.9^x^	58.4^x^	32.6^y^	0 ^z^	64.77*** (0.00)
Sufficient water for irrigation (%)	76.6	84.6^x^	71.9^y^	78.7^x^	72.7^xy^	5.12 (0.16)
Poor drainage (% of command area)	75.0	69.2^x^	65.7^x^	78.0^xz^	100^y^	25.07*** (0.00)
% Low lift pump (surface water)	65.5	69.2^x^	53.9^y^	66.7^x^	100^z^	34.13*** (0.00)
Pump owner and client-farmer live in same village (%)	96.6	98.1	96.6	96.1	97.7	0.73 (0.87)
% Sampled pump owner and client-farmers are relatives	34.5	34.6^xz^	35.9^x^	35.5^x^	22.7^z^	2.97 (0.40)
Pump owner and client-farmers pray in the same mosque/temple (%)	73.9	75.0 ^xyz^	69.7 ^x^	78.0 ^y^	63.6^xz^	6.56* (0.09)
Social relation index pump owner and client-farmer	0.0001	0.049^x^	−0.049^xz^	0.017^xy^	−0.130^z^	4.78 (0.19)
Irrigation service providers in the village (*n*)	42.7	67^x^	39^y^	45^y^	4 ^z^	92.08*** (0.00)

*Note*: *(**)[***] Means with diverging superscript letters across columns are statistically significantly different at the 10%(5)[1%] level of alpha error probability, based on multiple Mann-Whitney tests accounting for family-wise error; *P*-values in parentheses.

Pump owners who worked on a crop share basis served 78 farmers on average, each of whom cultivated 0.15 ha of *boro* rice. On average, the pump owner provided irrigation to 16 ha of land, and there were only four irrigation providers in the same village (Table 3). In contrast, other pump owners who worked on cash-based services managed relatively small command areas, smaller numbers of farmer-clients, who also had many alternative irrigation service providers (39–67) found in the same village (Table 3). These pump owners also used relatively lower horsepower pumps (Table 3). Interestingly, the client-farmers engaged in cash-based services cultivate larger *boro* on more land. Voluntary participation canal and drainage maintenance was not reported in share crop schemes and was only associated with cash-based services (33–58%, Table 3). LLPs were comprised 100% of the sample where crop-sharing was used, and 54–69%, where the cash payment was accepted (Table 3).

On average, farmers relying on hourly irrigation services paid BDT (Bangladesh Taka) 80 per hour^1^, comprising BDT 56 per hour for farmer-clients who provided fuel costs, and BDT 95 per hour for those who did not. In the 2013–14 *boro* season, the price of diesel – the major fuel of the irrigation pumps ‒ was BDT 70/l. The seasonal irrigation service payments averaged a cost of BDT 25,200/ha, but differed by modality (Table 4). The hourly irrigation service implied the lowest cost to farmer-clients (BDT 21,900/ha). Farmer-clients in the crop-sharing group paid the highest service charge –sharing on average 16.7% of their harvests with the pump owners, equivalent to BDT 32,300/ha^2^ (Table 4). Total seasonal *boro* production costs averaged BDT 90,800/ha for farmers, in which >27% cost was derived from irrigation.

**Table 4:** Average farmer irrigation expenditure, total production expenditure, pump owner revenue, and rice yields in Bangladesh, differentiated by irrigation service payment methods.

	All	Irrigation service				Kruskal-Wallis rank test Chi^2^ (overall differences)
		Hourly	Seasonal		Crop share	
			With client fuel	Without client fuel		
		a	b	c	d	(a ≠ b ≠ c ≠ d)
Client-farmers (*n*)	556	52	178	282	44	
Irrigation expenditure by client-farmers (BDT 000/ha)	25.2	21.93 ^x^	25.15 ^y^	24.73 ^x^	32.27 ^z^	107.71*** (0.00)
Total production costs (BDT 000/ha)	90.8	90.8	92.3	88.3	100.9	2.62(0.45)
Per ha fuel used for the entire season in 2013/14 (l)	401.3	397.8^x^	290.5^x^	507.5^x^	173.8^y^	22.3*** (0.00)
Cost of fuel/ha @ BDT 66.4/liter (000, BDT)	26.6	26.4 ^x^	19.3 ^x^	33.7 ^x^	11.5^y^	22.3*** (0.00)
Gross irrigation revenue, pump owner (BDT 000/ha)	17.8	9.28^x^	5.86^y^	24.73^z^	32.27 ^y^	122.04*** (0.00)
*Boro* rice yield (ton/ha)	6.59	7.14^x^	6.15 ^y^	6.86^x^	5.89^yz^	31.05*** (0.00)

*Note*: *(**)[***] Means with diverging superscript letters across columns are statistically significantly different at the 10%(5)[1%] level of alpha error probability, based on multiple Mann-Whitney tests accounting for family-wise error; *P*-values in parentheses.

In the case of six electric motors, we have divided the full season electricity bill by the average price of diesel BDT 66.4 to calculate per ha fuel costs.

On average, an irrigation engine consumed 401 l/ha of diesel equivalent fuel for an entire season for irrigation, however, the fuel consumption in crop-sharing arrangements was the lowest (174 l/ha, Table 4), reflective of the more common use of LLPs compared to STWs that are more energetically costly (Krupnik et al., 2015). Bangladesh is a net importer of crude oil and petroleum products. During 2016–18 (triennium average), Bangladesh imported 1.18 MMT of crude oil and 463,000 tons of refined petroleum products (BPC, 2018), at a value of worth of USD 3.38 billion (GoB, 2018). Although no solar irrigation pumps were found in our study, the greenhouse gas implications of fossil-fuel based irrigation relative to alternative power sources should be further studied. Table 4 also compares the (gross) irrigation service revenue to the pump owner – being highest for crop sharing. In contrast, the boro rice yields were lowest for crop share services and highest for hourly services (Table 4).

Sampled farmer-clients reported irrigation services were determined by local tradition (42%) and to a lesser extent by the pump owner (23%) in study areas (Table 5). However, in the case of crop sharing, survey respondents indicated that prior to the cropping season, farmers and pump owners would meet and decide the crop share percentage based on current fuel and paddy prices. Pump owners provided a much longer list of the factors that they consider in determining irrigation pricing, albeit a similar share acknowledged local tradition (47%). More commonly though, pricing decisions considered fuel price (83%), consultation with client-farmers (61%), or simply following other nearby pump owners’ pricing systems (55%). Some respondents elaborated that members of an irrigation scheme (pump owners and client-farmers) often meet before the beginning of the season to discuss and decide on irrigation service charges.

**Table 5:** Reported irrigation service determinants according to sampled client-farmers and pump owners, Bangladesh.

	All	Irrigation service				Kruskal-Wallis rank test Chi^2^ (overall differences)
		Hourly	Seasonal		Crop share	
			With client fuel	Without client fuel		
		a	b	c	d	(a ≠ b ≠ c ≠ d)
*Client-farmers’ view (%)*: Irrigation service determinants						
Client-farmers (*n*)	556	52	178	282	44	
Follow local tradition relating to irrigation water pricing	42	46^x^	42^x^	47^x^	9^y^	22.51*** (0.00)
Pump owner decides	23	23^x^	26^x^	24^x^	0^y^	14.24*** (0.00)
*Pump owners’ view (%):* Irrigation service determinants						
Pump owners (*n*)	139	13	44	71	11	
Consider fuel price	83	100^x^	74^y^	83^z^	100^x^	29.6*** (0.00)
Consult client-farmer	61	46^x^	56^x^	62^y^	91^z^	23.32*** (0.00)
Mimics price of neighboring pump owners	55	62^x^	54x	52x	73^y^	7.25* (0.06)
Follow local tradition relating to irrigation water pricing	47	38^x^	45^x^	48^x^	64^z^	6.81* (0.08)
Consider crop type	23	15^xz^	26^x^	16^x^	64^y^	52.67*** (0.00)
Consider soil type	14	23^x^	11^y^	17^x^	0^y^	13.58*** (0.00)
Distance of the field from the pump (meters)	20	46^x^	22^y^	16y^x^	0^z^	36.52*** (0.00)

*Note*: *(**)[***] Means with diverging superscript letters across columns are statistically significantly different at the 10%(5)[1%] level of alpha error probability, based on multiple Mann-Whitney tests accounting for family-wise error; *P*-values in parentheses.

On average, a sampled client-farmer had 4.6 years of formal schooling, 0.81 ha of land, and five family members. Seven percent were engaged in off-farm economic activities (Table 6). By contrast, pump owners were somewhat better endowed with 6.7 years of schooling, and 0.94 ha of land, an on average, more than five family members, although with similar (8%) engagement in off-farm income generation. Considering risk-taking profiles, on average, pump owners ranked themselves somewhat more risk-taking (mean score of 6.95 scores) than farmer-clients (6.21 score). Fifty-nine percent of pump owners reported at least one blood relative engaged in politics or governmental positions, against only 41% of the sampled farmers. Pump owners accepting crop share payments were endowed with less land (0.19 ha), though they were more risk-taking (risk score of 7.36) than pump owners in the other groups. Farmer-clients engaged in crop sharing were, in general, more resource constrained and risk-averse, and hence appear to be more interested in sharing risks with pump owners in the form of crop sharing agreement for irrigation water. In contrast, pump owners who opt for crop sharing are in general more risk-taking, and seemingly short on their own crop production or engaged in grain trading, and hence interested in in-kind produce payment as a compliment. These various variables are included in our empirical estimation procedures, discussed below.

**Table 6:** Selected descriptive statistics by the informant (sampled pump owner or client-farmer) by irrigation service, that can affect the irrigation service payment methods in Bangladesh.

	All	Irrigation service				Kruskal-Wallis rank test Chi^2^ (overall differences)
		Hourly	Seasonal		Crop share	
			With client fuel	Without client fuel		
		a	b	c	d	(a ≠ b≠c ≠ d)
*Client-farmers*						
Client-farmers (*n*)	556	52	178	282	44	
Years of schooling	4.59	4.79^x^	4.34^x^	4.64^x^	5.11^x^	1.34 (0.72)
% engaged in off-farm income generation	7.01	3.85^x^	3.93^x^	8.87^xy^	11.36^y^	6.14(0.11)
Total household members (*n*)	4.69	4.42^x^	4.59^x^	4.78 ^x^	4.77 ^x^	4.09 (0.25)
% of relatives in government job or politics	40.7	38.5^xy^	30.3 ^x^	46.8 ^yz^	45.5 ^xz^	12.78*** (0.01)
Land owned (ha)	0.81	0.73^xy^	0.90^x^	0.81^y^	0.54^z^	11.70*** (0.01)
Risk score	6.21	6.15 ^x^	6.26 ^x^	6.26 ^x^	5.82 ^x^	1.13 (0.77)
*Pump owner*						
Pump owners (*n*)	139	13	44	71	11	
Years of schooling	6.65	7.62 ^x^	6.28 ^x^	6.75 ^x^	6.36 ^x^	2.56 (0.46)
% engaged in off-farm income generation	7.91	15.38 ^x^	4.49 ^y^	8.51 ^x^	9.09 ^xy^	7.05* (0.07)
Total household members (*n*)	5.26	5.39^x^	5.23^x^	5.34 ^x^	4.73 ^z^	7.06* (0.07)
% of relatives in government job or politics	59.0	38.5^x^	55.1^y^	67.4^z^	45.5^xy^	21.69*** (0.00)
Land owned (ha)	0.94	0.23^xz^	0.30^xy^	1.59^xz^	0.19 ^z^	4.54 (0.21)
Risk score	6.95	6.85^x^	7.07^x^	6.84^x^	7.36^x^	2.25 (0.52)

*Note*: *(**)[***] Means with diverging superscript letters across columns are statistically significantly different at the 10%(5)[1%] level of alpha error probability, based on multiple Mann-Whitney tests accounting for family-wise error; *P*-values in parentheses.

## 5 Empirical findings: factors influencing irrigation service choice

We hypothesize the different irrigation services provide different incentive mechanisms to conserve water from farmer-clients’ point of view. Using the seasonal with client fuel service (a two-part tariff payment) as the default group in Table 7, we anticipated the hourly rate to provide the biggest incentive to conserve pump fuel use, which may be considered as a rough proxy for irrigation water, compared the seasonal flat rate without farmer’s fuel and crop sharing payments.

**Table 7:** Maximum likelihood estimates of multinomial logit models explaining the choice of payment method for irrigation services for service providers and client-farmers in Bangladesh.

	Dependent variable: irrigation service (seasonal with client fuel is base, = 0)					
	Service provider			Client-farmer		
Irrigation service	Hourly	Seasonal without client fuel	Crop share	Hourly	Seasonal without client fuel	Crop share
*Environmental domain variables*						
Service providers in village (*n*)	0.02** (0.01)	0.01 (0.00)	−0.24*** (0.08)	0.01*** (0.00)	0.004* (0.00)	−0.18*** (0.04)
Participation in voluntary community works dummy	−0.53 (0.69)	−1.31*** (0.46)	−20.4*** (1.14)	−0.18 (0.33)	−1.27*** (0.22)	−19.1*** (0.69)
Water availability during peak time season dummy	0.75 (0.93)	0.61 (0.54)	2.24* (1.32)	0.86** (0.43)	0.57** (0.25)	1.29** (0.58)
Poor drainage dummy	0.16 (0.76)	0.33 (0.48)	22.3*** (3.50)	−0.21 (0.33)	0.48* (0.25)	17.0*** (0.50)
*Human and social capital variables*						
Blood relative in government service or politics (dummy	−1.15 (0.85)	0.58 (0.51)	−1.37 (1.77)	0.19 (0.35)	0.58** (0.23)	0.30 (0.52)
Risk score	−0.005 (0.14)	−0.15 (0.10)	−0.04 (0.35)	−0.068 (0.08)	−0.087* (0.05)	−0.24** (0.11)
Years of schooling	0.12 (0.08)	0.04 (0.05)	0.20* (0.12)	0.04 (0.04)	0.02 (0.03)	0.11** (0.06)
Household members (*n*)	0.17 (0.16)	0.08 (0.12)	−0.21 (0.55)	−0.08 (0.14)	0.09 (0.07)	0.18 (0.15)
Major occupation in non-farm sector dummy	1.48 (1.14)	1.29 (0.97)	2.16 (2.05)	0.23 (0.80)	1.04** (0.45)	2.25** (0.93)
Land cultivated (ha)	−0.13 (0.42)	0.13*** (0.04)	−0.13 (0.11)	−0.37** (0.17)	−0.27** (0.13)	−1.47** (0.67)
Social relation index	0.21 (0.30)	0.29 (0.23)	−2.38* (1.37)	0.19 (0.18)	−0.035 (0.10)	−0.80** (0.37)
Northern district dummy	−2.21** (1.09)	−0.93* (0.52)	−4.00 (3.32)	−1.97*** (0.50)	−0.94*** (0.29)	−1.51** (0.72)
Constant	−2.81 (2.21)	0.27 (1.19)	−19.9*** (3.36)	−0.71 (0.90)	0.48 (0.60)	−14.6*** (1.14)

No. of observations		139			556	
Wald chi^2^(36)		1697.55***			3778.52***	
Pseudo *R*^2^		0.28			0.23	
Log pseudolikelihood		−113.41			−485.77	

*Note*: Values in parentheses are robust standard errors clustered at the respondent level. ***, ** and * indicate the 1%, 5%, and 10% levels of significance, respectively.

The number of irrigation service providers in a village is positively associated with the hourly service payment system for both pump owner and client-farmer, whereas the crop sharing method is strongly and negatively associated. The findings suggest that competition among irrigation service providers thereby encourages the adoption of relatively water conserving irrigation services. Voluntary participation in irrigation command area maintenance, which we used as a proxy for community relationship, is negatively associated with seasonal payment without farmer-client fuel supply systems, in addition to crop sharing irrigation services for both pump owner and client-farmer. This suggests that the presence of a strong community relationship discourages flat rate irrigation services that are less efficient directly in terms of fuel – and indirectly water.

The availability of irrigation water during the season was positively associated with crop sharing in the case of pump owners and all types of irrigation services (compared to two-part tariff methods) for client-farmers. The poor drainage dummy was positively associated with crop sharing for both the pump owner and client-farmer, reflecting problems with canal and field water management in lower-elevation areas of Barisal division (Krupnik et al., 2017). Poor floodwater drainage systems tend to make the irrigated *boro* rice farming riskier and less productive, with crop sharing allowing the partitioning of risks between farmers and pump owners while reducing the cash costs for the latter.

Having a blood relative in a government job or active in politics positively affected only the seasonal flat rate without fuel use by farmers. This could potentially be indicative of the farmers’ increased negotiation power. Where feasible, more risk-taking farmers likely prefer to reduce their reliance on seasonal flat rate without fuel, and crop sharing systems, which provide forms of risk sharing (e.g., Kajisa and Sakurai, 2005), and are therefore more favorably viewed by risk-averse farmers. Relatively more educated pump owners and farmers are more likely to prefer a crop sharing method. Farmers that however, derive most of their income from off-farm employment also appear to be more likely to choose crop sharing or the seasonal flat rate without fuel systems, possibly because they have higher assured opportunity costs. Interestingly, the size of pump owner’s landholdings was positively associated with the likelihood of using seasonal flat rate payments without fuel, whereas client-farmer farm size was negatively correlated with all irrigation services compared to the two-part base tariff. Well-off pump owners are economically more capable of bearing irrigation expenses including the cost of fuel for an entire season; conversely, economically affluent client-farmers are more likely to choose the two-part tariff, in which farmers can be the residual claimer after paying the irrigation charge.

Finally, the social relation index between pump owner and farmers has negatively associated the likelihood of crop sharing, suggesting that social bondage can increase incentives to opt for socially desirable payment systems, but without such capital, less risky and potentially more cost-effective irrigation services may prevail. Compared to the two-part tariff payment as a base, the hourly, seasonal flat rate and crop sharing payment systems are less prevalent in northern or western Bangladesh, where civil infrastructure is relatively more developed with greater cropping intensity (Mottaleb et al., 2016).

## 6 Conclusions and policy recommendations

Bangladesh’s privatization policies and the liberalization of machinery imports contributed to the rapid proliferation of small-scale mechanized pumps and irrigation services provision. Using primary information collected from 556 farmers and 139 pump owners, this study demonstrates that different irrigation service payment systems have emerged in Bangladesh over time, with a shift away from crop sharing towards cash-based options. This study unpacks the development of varying types of pricing systems and explores the factors that contributed to their differentiation.

Today, irrigation in Bangladesh primarily relies on groundwater abstraction using a diesel engine and centrifugal pump, with a significant environmental footprint. Currently, out of 5.3 million ha of the total irrigated land, 58% is irrigated by 1.31 million diesel engines (BADC, 2015). On average, 401 l of diesel were used to irrigate one ha of *boro* rice land during the 2013–14 winter season. This equates to roughly 1.23 billion liters of diesel being consumed for in a single season. Stationary burning of one liter of diesel results in the release of 2.69 kg of direct and embodied carbon dioxide (CO_2_) (IPCC, 2006). In a single *boro* season, Bangladesh thus produces 3.30 billion kg CO_2_ from burning diesel for irrigation alone. Actual emissions from rice cropping are set to be even higher when accounting for pump efficiency losses, groundwater depth, and considering emissions of CH_4_ and N_2_O from soil-water based processes related to agronomic management practices. Policy may want to explore alternative irrigation modalities – including increased rural electrification, electric motors, solar irrigation options, and more fuel-efficient diesel pump alternatives that – when appropriately managed – could help mitigate these externalities.

Our study demonstrates that Bangladesh’s irrigation payment systems are mainly groundwater-based, with traditional centrifugal pumps used for water abstraction. Recently, projects led by the International Maize and Wheat Improvement Center (CIMMYT) have introduced axial flow pumps (AFP) that are suitable for surface water irrigation and respond to governmental policy priorities championing increased surface water use (Krupnik et al., 2015; Mottaleb, 2018). AFPs can lift from 72% to 55% more water at 1 and 2 m lifts, respectively, compared to centrifugal LLPs, with 51% and 21% greater fuel efficiency, respectively (Mottaleb, 2018; Valle et al., 2014). Currently, roughly 173,000 centrifugal LLPs are engaged in irrigating 1.16 million ha of land using surface water. Rapid replacement of the LLPs by AFPs cold help to mitigate some of the negative environmental ramifications of irrigation while offering pump owners and farmer-clients opportunities for fuel and overall payment cost reductions, respectively. Where appropriate, AWD can also be attempted (Qureshi et al., 2015), although evidence in Bangladesh indicates new irrigation pricing structures and business models that align pump owner and farmer-clients interests – for example, volumetric-based payment systems – may be needed to encourage widespread adoption (Pearson et al., 2018). Other novel pricing systems are also being experimented with at this time, including pay-as-you-go DTW irrigation system that equates with volumetric water pricing using smart-cards and automatic payment machines located at the pump source, although this system remains in a pilot stage and is used only in select areas of northwest Bangladesh (Islam, 2016). These topics are similarly relevant to the irrigated systems that prevail in other South Asian countries.

The study confirms that geography and pump type can influence the irrigation service choice, in which farmer-clients and pump owners in relatively riskier environments, such as Barisal division are more likely to choose crop sharing. Social capital and risk profiles also substantially influence irrigation payment types. Public support reducing crop production risks could potentially encourage risk-averting farmers to choose more socially desirable water conserving irrigation services, such as hourly or two-part tariff methods that both constitute a variable production cost and, hence, provide an incentive to reduce the quantity of water applied. Finally, our findings indicate that competition among pump owners could contribute to the emergence of socially desirable irrigation services with an in-built incentive for water conservation. These hypotheses, however, need to be verified with further research. A major policy recommendation, therefore, will be how to encourage and maintain healthy competition among service providers to enhance smallholder adoption of sustainable and environmentally sounds irrigation practices alongside increased food security given Bangladesh’s pursuit of the Sustainable Development Goals.

## Figures and Tables

**Figure:**
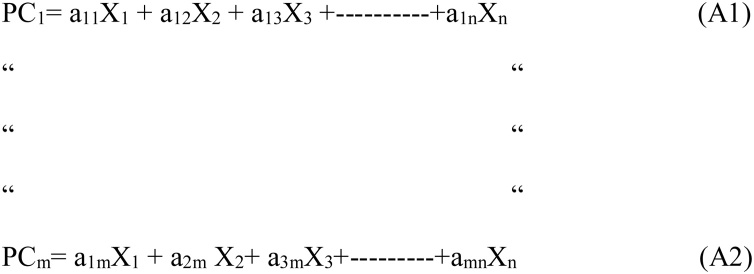


**Table A1:** Summary results from Principal Component factor analysis.

Factor	Dummy variable	Eigenvalue	Unique variances	Kaiser-Meyer-Olkin measure of sampling adequacy
Factor 1	Pump owner and client-farmer are relative	1.48	0.61	0.50
Factor 2	Pump owner and client-farmer pray in the same mosque/temple	0.95	0.31	0.50
Factor 3	Pump owner-client-farmer live in same village	0.58	0.61	0.50
Overall				0.50
Observations (*n*)				556
Retained factors				1
Parameters (*n*)				3

This social relation index was used in our analysis to assess if the social relationship between pump owners and client-farmers affects the structure of irrigation water pricing method and the amount of payment made by client-farmers to the pump owners. We assume that client-farmers with more social interaction with pump owner (indicative of social capital and measured by higher social indices) are more likely to choose mutually beneficial and socially acceptable payment methods and rates.

## Appendix A Social relation index calculation

Principal component analysis (PCA) was used to generate the relation indices for each household based on information regarding whether or not the pump owner is a blood relative (brother, sister or first cousin) of their client-farmers (yes = 1, or 0 otherwise), a dummy for whether or not client-farmer and the pump owner live the same village (yes = 1, or 0 otherwise), and a dummy for whether or not client-farmer and pump owner pray in the same mosque or temple (yes = 1, or 0 otherwise). While the importance of social interactions and repeated transactions in mitigating market failure is often recognized in the literature following the seminal papers of Arrow (1968) and Otsuka and Hayami (1988), the application of social bonds and relations in influencing irrigation water pricing method and payment has not been previously explored.

PCA is a multivariate procedure used to reduce dimensions of a dataset by aggregating similar variables through orthogonal liner combination. Mathematically, from an initial set of *n* correlated variables, PCA creates orthogonal components, where each component is a linear weighted combination of the original variables. For *n* social interaction variables between pump owner and client-farmer, for instance,

where *a_mn_* represents the weight for the *m*^th^ principal component and the *n*^th^ variable.

The weights for each principal component PC are given by the eigenvectors of the covariance matrix shown in Table A1. Using first PC scores and the mean and standard deviation of the original dataset, social relation indices were computed as follows:

(A3) $$W_{j}=\sum_{i}^{n}[\gamma_{i}*(X_{ij}-\bar{Xi})/\delta_{i}]$$

where *W_j_* is the relation index sampled farmer-clients, $\gamma_{i}$ represents the weights (scores) assigned to the *n* indicators of social interaction between pump owners and client-farmers on the first PC, $X_{ij}$ is the original observation of social interaction between pump owner *i* and farm household *j*, $\bar{Xi}$ is the mean number of the sampled indicators related to social interactions for the *i^th^* farm household of each of the *n* variables, and $\delta_{i}$ is the standard deviation of each of the social interaction variables in the sample.

## Footnotes

1 USD 1 =  BDT 83 (approximately).

2 Calculated based on the average yield per hectare of 5.89 tons, and the price of rice at harvest per 40 kg was BDT 600.
